# Acute and Recurrent Pancreatitis in Children: Insights into Etiology and Clinical Course from a Retrospective Single-Center Study

**DOI:** 10.3390/children13081084

**Published:** 2026-08-15

**Authors:** Alexandra Mititelu, Alina Grama, Gabriel Bența, Alexandru-Ștefan Niculae, Tudor Lucian Pop

**Affiliations:** 1 Second Pediatric Discipline, Department of Mother and Child, Iuliu Haţieganu University of Medicine and Pharmacy, 400177 Cluj-Napoca, Romania; perta.alexandra@elearn.umfcluj.ro (A.M.); benta.gabriel@elearn.umfcluj.ro (G.B.); tudor.pop@umfcluj.ro (T.L.P.); 2 Second Pediatric Clinic, Center of Expertise in Pediatric Rare Liver Diseases, Emergency Clinical Hospital for Children, 400177 Cluj-Napoca, Romania

**Keywords:** pediatric pancreatitis, acute recurrent pancreatitis, etiology, hypoalbuminemia, hospitalization length

## Abstract

**Highlights:**

**What are the main findings?**

- The etiological profile of pediatric pancreatitis differed significantly between first-episode and recurrent disease: genetic causes accounted for more than one-third of ARP cases, whereas idiopathic and infectious etiologies predominated in first-episode AP.
- Etiological complexity at first presentation, rather than recurrence status, emerged as a significant predictor of prolonged hospitalization (Welch ANOVA *p* = 0.007), while admission hypoalbuminemia was significantly associated with moderate-to-severe disease.

**What are the implications of the main findings?**

- These findings support a stepwise diagnostic approach in which comprehensive etiological work-up, including genetic testing, is reserved for children with recurrent pancreatitis, rather than pursued exhaustively after a first, often self-limited episode.
- Two practical, widely accessible markers can support early clinical decision-making: etiological complexity at first presentation identifies children likely to require prolonged hospitalization, and admission serum albumin serves as a simple, low-cost indicator of more severe disease.

**Abstract:**

**Background/Objectives:** Pediatric acute pancreatitis (AP) is increasingly recognized as a clinically significant disease, yet Central and Eastern European cohort data remain limited. Acute recurrent pancreatitis (ARP) affects a substantial proportion of these children and may reflect distinct underlying etiologies. This study aimed to characterize the etiological spectrum, disease severity, and hospitalization outcomes of AP and ARP in a pediatric tertiary referral population, and to identify early clinical predictors of severity. **Methods:** We retrospectively analyzed 63 children hospitalized between 2018 and 2025 with 77 documented episodes of AP. Diagnosis followed INSPPIRE criteria, and severity was graded using the 2017 NASPGHAN classification. Etiologies, clinical presentation, laboratory parameters, imaging findings, and hospitalization length were compared between AP and ARP groups, across severity and etiological complexity categories using appropriate non-parametric and permutation-based methods. **Results:** Genetic etiologies predominated in ARP (37.0%), whereas idiopathic and infectious causes were more common in first-episode AP (24.0% and 14.0%, respectively; overall *p* < 0.001). Severity distribution did not differ between AP and ARP, with mild disease accounting for the majority of episodes in both groups. Serum albumin was significantly lower in moderate/severe episodes (*p* = 0.030). Within the single-episode subgroup, etiological complexity emerged as a significant predictor of prolonged hospitalization, with complex multifactorial or systemic etiologies associated with markedly longer stays than idiopathic or single-factor disease (Welch ANOVA *p* = 0.007). **Conclusions:** In this Romanian pediatric cohort, genetic causes dominate ARP, while idiopathic and infectious etiologies characterize first-episode AP, supporting a stepwise approach in which comprehensive etiological work-up, including genetic testing, is prioritized after recurrence. The predominance of genetic causes in ARP should be interpreted with caution, as genetic testing was applied selectively, predominantly after recurrence. Recurrence status alone does not predict severity, whereas etiological complexity at first presentation and hypoalbuminemia represent practical, accessible early markers for clinically assessing more severe disease.

## 1. Introduction

Acute pancreatitis (AP) in children is defined according to the consensus framework of the International Study Group of Pediatric Pancreatitis: In Search for a CuRE (INSPPIRE) consortium [1]. Once considered rare, pediatric AP is now diagnosed substantially more often than in previous decades, with incidence estimates of roughly 1 case per 10,000 children each year and regional figures between 2 and 13 cases per 100,000 children annually [2]. A recent analysis of the Global Burden of Disease 2021 database confirmed that pancreatitis remains a significant cause of morbidity in children and adolescents worldwide, with marked regional variation and an age-dependent pattern [3]. Beyond its rising incidence, pediatric AP carries a substantial clinical and healthcare burden: episodes frequently require hospitalization, and moderate-to-severe disease is associated with prolonged length of stay and increased resource utilization. Data specific to Central and Eastern Europe, however, remain scarce.

The etiology of AP in children differs substantially from that in adults and varies by age, geographic region, and diagnostic practice, encompassing biliary, traumatic, infectious, drug-induced, metabolic, systemic, anatomical, and genetic causes. The predominant etiology varies markedly by region, with gallstones predominating in Asia, trauma in Oceania, and idiopathic disease in Europe, North America, and South America [4].

The clinical course of pediatric AP is generally favorable, with most children experiencing a single, self-limited episode. However, a clinically important subset progresses to acute recurrent pancreatitis (ARP). Pooled data from a 2025 meta-analysis indicate that approximately 20% of children who experience a first AP episode will have at least a second, and that progression to chronic pancreatitis (CP) occurs in more than one third of these, with the initial episode’s etiology and severity acting as the principal determinants; episodes linked to genetic or structural/anatomical causes carry a markedly higher downstream risk than those linked to infection or toxin exposure [5].

Pediatric pancreatitis is thus diagnosed using the INSPPIRE criteria [1]. A child is considered to have AP once any two of three elements are documented: a compatible clinical pain pattern, a threefold or greater rise in serum amylase and/or lipase above the reference range, or radiologic findings of AP. Two or more distinct episodes separated by a pain-free interval with enzyme normalization define ARP, whereas persistent imaging or functional evidence of irreversible pancreatic damage defines CP. When imaging is needed to confirm the diagnosis or characterize complications, abdominal ultrasound is the preferred initial modality in children because it avoids ionizing radiation and is widely accessible, while computed tomography and magnetic resonance cholangiopancreatography are reserved for uncertain or complicated cases [6].

Considering the substantial clinical and resource burden of pediatric AP and ARP, and the scarcity of Central and Eastern European cohort data, the aim of this study was to characterize the etiological spectrum, disease severity, and hospitalization outcomes of AP and ARP in a pediatric tertiary referral population from this region, and to identify early, widely accessible clinical and laboratory predictors of disease severity and prolonged hospitalization.

## 2. Materials and Methods

We retrospectively analyzed children hospitalized at the 2nd Pediatric Clinic of the Emergency Clinical Hospital for Children, Cluj-Napoca, Romania, between 2018 and 2025 who had a diagnosis of AP or ARP. The inclusion criteria were at least one episode of AP, age below 18 years, and formal consent from the patient and/or legal guardian. The exclusion criteria were incomplete data available or age above 18 years. Traumatic pancreatitis was not represented in this cohort, as no cases of trauma-associated AP occurred during the study period.

Data were collected from hospital medical records and included demographic and clinical information such as age, sex, primary and secondary diagnoses, clinical manifestations, complications, laboratory findings, imaging results, and treatment. The diagnosis of AP was established according to the INSPPIRE criteria. The threshold for pancreatic enzymes was set at our laboratory’s reference values: 100 UI/L for amylase and 60 UI/L for lipase. Laboratory parameters were obtained at admission and reassessed at 24–48 h, with further sampling during hospitalization as clinically indicated. Genetic analysis was performed using a targeted pancreatitis gene panel (Blueprint Genetics, Espoo, Finland). Testing was applied selectively rather than to all patients, most often following a recurrent episode, consistent with a stepwise diagnostic approach.

Severity of AP was classified as mild, moderate, or severe based on the 2017 NASPGHAN classification. Mild AP was defined by the absence of local or systemic complications and the absence of organ dysfunction. Moderate AP included cases with local and/or systemic complications and/or organ dysfunction persisting for less than 48 h. Severe AP (SAP) was defined by organ dysfunction lasting longer than 48 h [7]. Local complications, as described by Abu El-Haija and colleagues [7], included peripancreatic fluid collections, acute necrotic collections, pancreatic or peripancreatic necrosis (sterile or infected), pseudocysts, and walled-off necrosis (sterile or infected). Systemic complications comprised multiple organ dysfunction and the requirement for intensive care unit (ICU) admission. Organ dysfunction was defined according to the criteria established by the International Pediatric Sepsis Consensus Conference in 2002 [8].

Statistical analysis was performed using JASP software (Version 0.97, University of Amsterdam, Amsterdam, The Netherlands). Continuous variables were tested for normality using the Shapiro–Wilk test. As most variables showed non-normal distributions, continuous data were expressed as medians and interquartile ranges (IQRs). Categorical variables were presented as frequencies and percentages.

Comparisons between patients with AP and ARP were performed using the Mann–Whitney U test for continuous variables and the Chi-square test or Fisher’s exact test, as appropriate, for categorical variables.

To evaluate factors associated with disease severity, episodes were classified into two groups: mild acute pancreatitis and moderate/severe acute pancreatitis. Continuous variables were compared using the Mann–Whitney U test, while categorical variables were analyzed using the Chi-square test or Fisher’s exact test. For the single-episode subgroup analysis (Section 3.4), etiological factors were grouped into six categories: idiopathic, biliary, infectious, multifactorial, toxic, and other. Associations between etiological category and categorical outcomes (sex, age group, disease severity) were evaluated using the chi-square test with Monte Carlo permutation (10,000 resamples), given small expected cell frequencies. This approach was applied both to the primary etiological comparison between AP and ARP groups (Section 3.2) and to the single-episode subgroup analyses (Section 3.4).

Differences in age across etiological groups were assessed with the Kruskal–Wallis test. Differences in hospitalization length across six etiological groups were evaluated using one-way ANOVA; for the consolidated three-group comparison (idiopathic, single-factor, and complex etiologies), a Welch one-way ANOVA was applied due to heteroscedasticity (Levene *p* = 0.047), with Games–Howell post hoc testing. A non-parametric Kruskal–Wallis test was additionally performed to confirm the result without reliance on distributional assumptions. A two-sided *p*-value < 0.05 was considered statistically significant.

The study was approved by the Ethics Committee of the Iuliu Haţieganu University of Medicine and Pharmacy, Cluj-Napoca (No. 112/21 June 2023).

## 3. Results

### 3.1. Characteristics of the Study Group

Our study included 63 children with 77 episodes of AP. Our cohort comprised two groups of patients: 50 children with only one episode of AP and 13 children with 27 episodes of AP, categorized as ARP.

The recurrence rate for our patients was 20.6%. Girls represented 57.1% of all cases. Abdominal pain was the most representative symptom, present in 97.4% of all acute episodes, and was mostly diffuse or epigastric. Other symptoms included nausea 42.9%, loss of appetite 80.5%, vomiting 62.3%, jaundice 15.6%, diarrhea 22.1% and fever 16.9%.

Abdominal US was the primary imaging modality. Only a small percentage of patients had CT scans or MRCP: 15.58% and 10.38%, respectively. These investigations were performed in acute episodes with severe local or systemic complications. CT was therefore reserved for selected complicated cases and not performed across the full severity spectrum. Local complications observed in our patients were mostly peripancreatic fluid collections (23.37%) and pseudocyst formation (one case).

**Figure 1:**
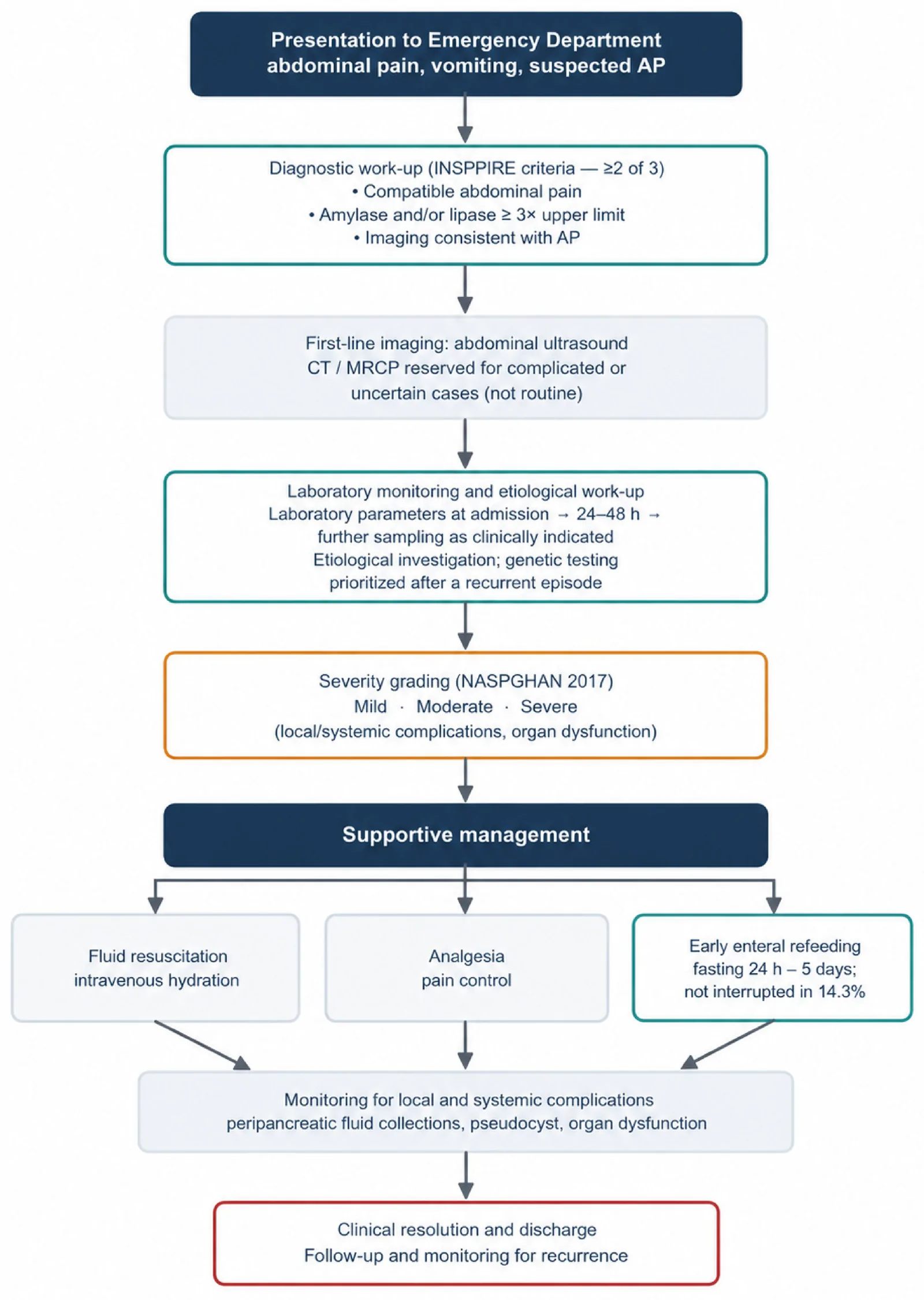
Diagnostic and therapeutic pathway of pediatric AP.

The treatment of acute episodes was mostly supportive and was similar in both groups, the single episodes and the recurrent ones. Fluid administration and pain medication were the most important measures taken in each case. Stopping oral feeding lasted between 24 h and 5 days. In a small percentage of patients, the oral feeding was not stopped at all (14.28%). Feeding management prioritized early enteral refeeding rather than routine nasogastric drainage or parenteral nutrition. Reintroduction of feeding was guided by clinical tolerance rather than enzyme normalization, and the short duration of fasting meant the conventional indication for parenteral nutrition did not arise. A flowchart showing the pathway for investigations and treatment of our patients is presented in Figure 1.

A comparison of demographic and laboratory parameters between AP and ARP patients is presented in Table 1. No statistically significant differences were observed between the two groups in age or length of hospitalization. Regarding biological parameters, no statistically significant differences were observed in leukocyte count, lymphocyte count, liver enzymes, bilirubin, triglycerides, pancreatic enzymes, C-reactive protein (CRP) levels, or procalcitonin (all *p* > 0.05). Alanine aminotransferase (ALT) and gamma-glutamyl transpeptidase (GGT) serum levels tended to be higher in the AP group. However, these differences did not reach statistical significance (*p* = 0.058 and *p* = 0.061, respectively).

### 3.2. Etiologies of AP and ARP

The distribution of etiological factors differed significantly between patients with AP and those with ARP (χ^2^(6) = 25.22, *p* < 0.001; Monte Carlo *p* = 0.0001, based on 10,000 permutations). Given that 50% of expected cell frequencies were below 5, a Monte Carlo simulation was applied to confirm the validity of the asymptotic result. The etiologies were divided into seven categories: idiopathic, infectious, toxic, genetic, biliary, multifactorial, and other. The “other” category (*n* = 17 episodes) comprised systemic diseases (*n* = 7, predominantly inflammatory bowel disease), metabolic diseases (*n* = 6, including hypertriglyceridemia, diabetic ketoacidosis, and cystic fibrosis), and pancreatic malformations (*n* = 4, pancreatic divisum). The “multifactorial” category (*n* = 9 episodes) comprised cases with more than one concurrent etiological factor, most commonly combinations of infection, drug/toxic exposure, and an underlying systemic or hematological condition. Genetic testing was performed selectively, most often after a recurrent episode, using a targeted pancreatitis gene panel. Among the patients tested, pathogenic or likely pathogenic variants were identified in the *CFTR*, *SPINK1*, *PRSS1*, and *CTRC* genes, across both first-episode and recurrent presentations. Genetic etiologies accounted for a single first-episode AP case and for the majority of recurrent cases. The frequency of each etiology is shown in Table 2.

Genetic etiologies were markedly more frequent among patients with ARP, accounting for 37% of recurrent cases compared with only 2% of first-episode AP cases. In contrast, idiopathic pancreatitis was substantially more common in the AP group (24% vs. 3.7%), while infectious etiologies were observed exclusively in patients with AP.

Biliary pancreatitis showed a similar distribution across groups, accounting for 16% of AP cases and 14.8% of ARP cases. Likewise, the proportions of multifactorial and toxic etiologies were comparable between the two groups. The category of “other” etiologies was slightly more frequent among ARP patients, although this difference was less pronounced than that observed for genetic and idiopathic causes.

### 3.3. Severity Analysis

**Figure 2:**
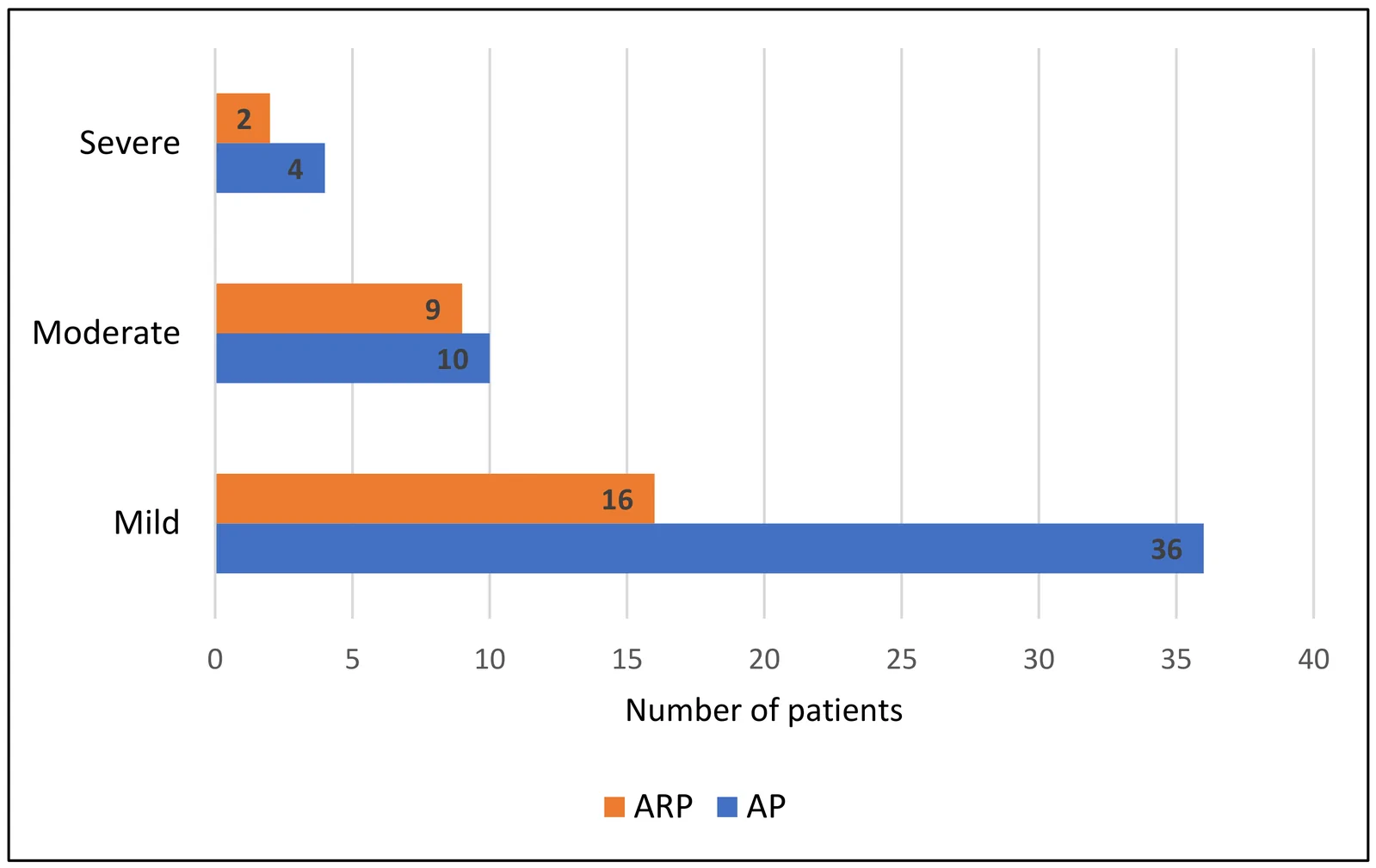
Severity distribution in acute pancreatitis (AP) and acute recurrent pancreatitis (ARP).

In all patients, AP and ARP were stratified by severity according to the NASPGHAN classification. In the single-episode group, the severity distribution showed mostly mild cases (72%), followed by moderate (20%) and severe (8%). In the ARP group, the severity distribution was similar, with 59.25% mild, 33.33% moderate, and 7.40% severe forms. As illustrated in Figure 2, mild pancreatitis represented the predominant clinical presentation in both the AP and ARP groups. Although moderate pancreatitis appeared more frequently among patients with recurrent disease, the overall distribution of severity categories did not differ significantly between AP and ARP (χ^2^(2) = 1.69, *p* = 0.429). Severe pancreatitis was uncommon in both groups.

The patients were afterward grouped as mild and moderate/severe acute pancreatitis, because of the small number of severe cases. Comparisons of demographic, clinical, and laboratory characteristics according to disease severity are presented in Table 3.

Patients with moderate/severe pancreatitis experienced significantly longer hospital stays than those with mild disease, with a median hospitalization duration of 7 days (IQR 8) compared with 5 days (IQR 4.25) in the mild group (*p* = 0.005). This finding reflects the increased clinical burden and resource utilization associated with more severe episodes of pancreatitis.

Serum albumin levels were significantly lower among patients with moderate/severe disease. The median albumin concentration was 3.75 g/dL (IQR 1.13) in the moderate/severe group, compared with 4.10 g/dL (IQR 0.83) in patients with mild pancreatitis (*p* = 0.030). This observation suggests that hypoalbuminemia may be associated with a more severe inflammatory response and could potentially serve as an indicator of disease severity.

No statistically significant differences were observed regarding age, leukocyte count, lymphocyte count, liver enzymes (AST, ALT, GGT), total bilirubin, triglycerides, lipase, CRP at admission, or procalcitonin levels (all *p* > 0.05).

Serum amylase and 24–48 h CRP were numerically higher in the moderate/severe group, but these differences were not statistically significant and should be regarded as hypothesis-generating observations, most plausibly limited by the small number of severe episodes rather than indicating a true association.

### 3.4. Single Episode Analysis

The single-episode AP group comprised 50 pediatric patients. Etiologies were classified into six categories: idiopathic (*n* = 12), biliary (*n* = 8), infectious (*n* = 7), multifactorial (*n* = 7), toxic (*n* = 6), and other (*n* = 10).

The distribution of etiological categories did not differ significantly by sex (χ^2^(5) = 2.716, Monte Carlo *p* = 0.763) or by age group (1–5, 6–10, >11 years; χ^2^(10) = 8.239, Monte Carlo *p* = 0.640). Comparison of age distribution across the six etiological categories revealed no statistically significant differences (Kruskal–Wallis H(5) = 6.301, *p* = 0.278), although patients with infectious pancreatitis tended to be younger (median 8.0 years) and those with biliary disease were older (median 13.5 years).

**Figure 3:**
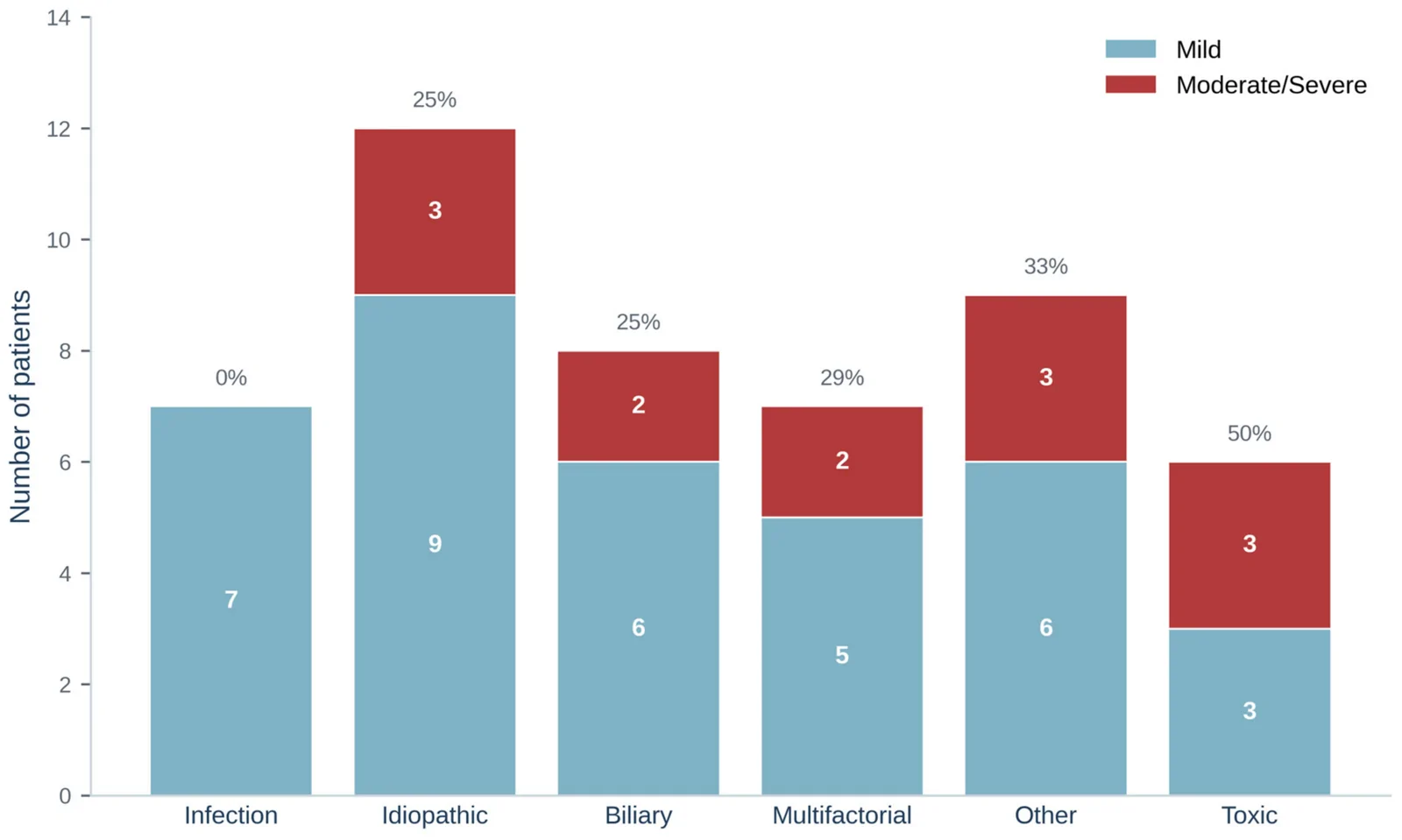
Disease severity by etiological category in the single-episode acute pancreatitis subgroup. Bars show the number of patients classified as mild or moderate/severe within each of the six etiological categories; percentages above each bar indicate the proportion of moderate/severe disease.

No significant association was found between etiological category and disease severity (mild versus moderate/severe; χ^2^(5) = 4.967, Monte Carlo *p* = 0.450), despite descriptively higher proportions of moderate-to-severe disease in the toxic (50.0%) and other (40.0%) groups (Figure 3).

Mean hospitalization length ranged from 5.42 ± 2.54 days (idiopathic) to 10.14 ± 4.22 days (multifactorial). One-way ANOVA across the six etiological categories showed a non-significant trend toward longer stays in certain groups (F(5, 44) = 2.328, *p* = 0.058).

**Figure 4:**
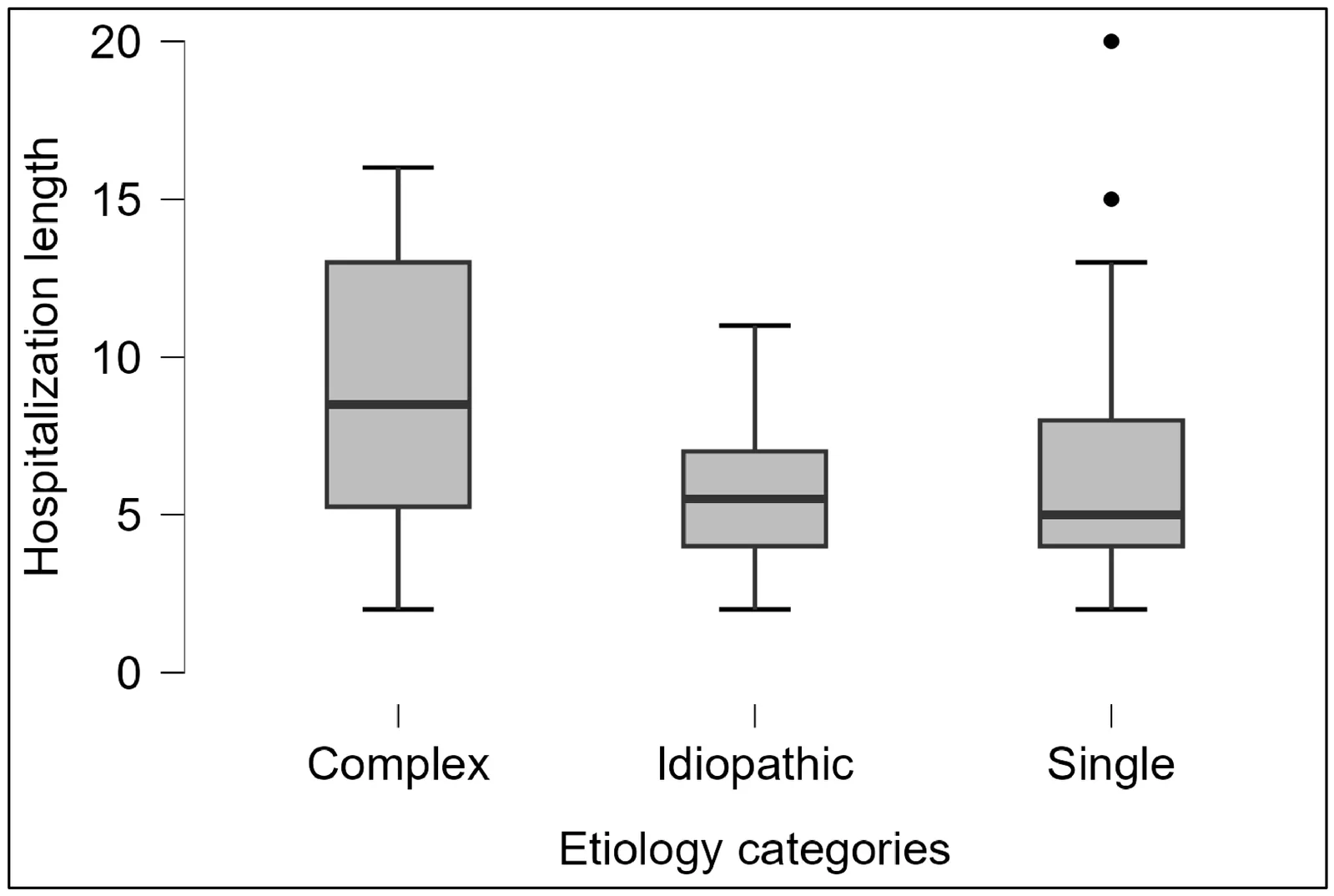
Hospital length of stay (days) by etiological complexity in pediatric acute pancreatitis.

Etiologies were further consolidated into three complexity-based groups: idiopathic, single-factor (biliary, infectious, toxic, malformations, genetic), and complex (multifactorial, systemic, and metabolic diseases). A Welch one-way ANOVA revealed a significant difference in length of hospitalization across these groups (F(2, 24.33) = 6.222, *p* = 0.007) This finding was confirmed by a non-parametric Kruskal–Wallis test (H(2) = 10.98, *p* = 0.004), indicating that the association between etiological complexity and hospitalization length does not depend on parametric assumptions. Patients with complex etiologies had the longest stays (mean 9.93 ± 4.10 days), compared with those with single-factor etiologies (5.96 ± 2.66 days) and idiopathic etiologies (5.42 ± 2.54 days). Games–Howell post hoc testing confirmed significantly longer hospitalization for complex versus idiopathic etiologies (mean difference 4.51 days, *p* = 0.002) and for complex versus single-factor etiologies (mean difference 3.97 days, *p* = 0.004); the idiopathic and single-factor groups did not differ significantly (*p* = 0.559), as illustrated in Figure 4.

## 4. Discussion

### 4.1. Demographic and Epidemiological Context

The present cohort showed a slight female predominance and a recurrence rate of approximately one in five cases. Both findings sit within, though toward the upper end of, the published range. In the systematic review by Juhász et al. of 44 pediatric AP cohorts, the proportion of female patients varied widely (37–73.3%), with most studies clustering around 48–60% [9]. With respect to recurrence, the 48-study meta-analysis by Tian et al. reported pooled recurrence rates of 15.3% in North America, 13.1% in Asia, and 13.8% in Europe [4]. The more recent 2025 pooled analysis by Gagyi et al. focused specifically on AP-to-ARP-to-CP progression, converged on a similar overall figure, close to 20%, for recurrence after a first AP episode [5], a value that aligns almost exactly with the recurrence observed in the present cohort. This convergence with the most recent and comprehensive pooled estimate available suggests that the recurrence rate identified here, while at the higher end of older regional estimates, is well within the range expected from contemporary international data.

### 4.2. Etiology of AP and ARP

The most important etiological finding of the present study is a significantly different distribution between AP and ARP, driven by a marked predominance of genetic causes in ARP and of idiopathic and infectious causes in first-episode AP. These results closely parallel the central finding of the INSPPIRE consortium, the largest international collaborative effort in this field [10]. Within the INSPPIRE-2 cohort, CP was distinguished from ARP largely by an enrichment of genetic and obstructive risk factors, whereas ARP cases were more often linked to genetic mutations, toxic-metabolic triggers, and elevated triglycerides [11]. The present results reinforce this pattern in an independent, geographically distinct cohort and support prioritizing genetic testing after a second episode of pancreatitis rather than after the first.

The proportion of biliary etiology in the present cohort was lower than in several other reported series. In a single-center cohort from Western China, biliary disease accounted for 31.5% of cases and, together with idiopathic disease (28.5%), was the leading etiological category [12].

A particularly relevant and recently published comparison cohort is a large Canadian study. Across 208 children with AP followed between 2014 and 2021, drug-induced disease emerged as the single most frequent cause, accounting for roughly one in five cases (20.2%); biliary disease (16.4%) and infectious triggers (15.4%) ranked second and third [13]. The 16.4% biliary figure in that cohort sits close to the biliary proportion observed in both the AP and ARP groups of the present study, whereas the comparatively high drug-induced proportion in the Canadian cohort, nearly double the toxic/drug-related proportion observed here, may reflect a tertiary-referral case-mix with greater exposure to chemotherapeutic and other high-risk medications.

Two recently published pediatric cohorts provide additional external context for these etiological findings. A Polish single-center cohort of 57 children grouped causes of AP into four categories: idiopathic disease was the largest single group (35.1%), followed by biliary causes (24.6%) and a combined category of ductal mutations and/or anatomical pancreatic defects (24.6%), with infectious causes accounting for the remainder (10.5%). Within that same cohort, 19 of the 57 children (33.3%) experienced more than one episode of AP during the study period, a recurrence rate even higher than the one observed here, though derived from a smaller sample [14]. In a larger Chinese cohort of 102 children, idiopathic disease again topped the list (28.4% of cases), with traumatic (25.5%) and biliary (23.5%) causes close behind, and anatomical abnormalities (15.7%) and drug-induced injury (6.9%) accounting for the remaining cases [15]. The convergence of idiopathic disease as the single most common, or one of the most common, etiological categories across multiple independent, recently published (2025–2026) cohorts from North America, Central Europe, and East Asia, despite substantial differences in population genetics and healthcare infrastructure, lends additional external validity to the comparably high idiopathic proportion identified in first-episode AP in the present cohort.

Two additional cohorts further illustrate the geographic heterogeneity of pediatric AP etiology. In a large Chinese 10-year retrospective study of 275 children (330 episodes), biliary tract disease and viral infection emerged as the dominant etiological categories, with severe AP accounting for 10.2% of episodes [16], a substantially higher proportion than in the present cohort reflecting the well-established Asian pattern of biliary-predominant pediatric pancreatitis documented in broader meta-analytic data [4]. By contrast, a North African single-center series from Morocco identified idiopathic disease as the leading etiology (26.6%), followed by traumatic and biliary causes in equal proportions (20.0% each) [17], a distribution closely mirroring the pattern observed in the present Romanian cohort and consistent with the European and non-Asian predominance of idiopathic disease reported across the literature. Taken together, these findings reinforce that the etiological profile of pediatric AP is substantially shaped by geographic and demographic context, and that the relatively high idiopathic proportion observed in the present study is not an artifact of cohort size or referral pattern, but reflects a reproducible regional pattern.

### 4.3. Clinical Presentation, Imaging, and Treatment

Abdominal pain was nearly universal in the present cohort, consistent with its established role as the cardinal presenting symptom of pediatric AP. In a Bahraini series, all 56 enrolled children underwent an abdominal US, while only a minority underwent more advanced imaging (16.1% had a CT scan and 8.9% an MRCP) [18]. This diagnostic pathway closely mirrors the imaging strategy reported in the present study and is consistent with current recommendations favoring US as the first-line, radiation-free imaging modality in children [6]. The preference for early, even immediate, enteral feeding in a subset of patients (14.28% of episodes managed without interruption of oral intake) is in line with the broader shift in pediatric AP management toward early refeeding, supported by US national data showing that prolonged fasting and reliance on parenteral nutrition are independently associated with longer hospitalization rather than improved outcomes [19].

### 4.4. Disease Severity

Using the 2017 NASPGHAN classification, the present cohort showed a predominant proportion of mild disease in single-episode AP and in ARP, with no statistically significant difference in severity distribution between the two groups. This is broadly in line with the literature: in the systematic review by Juhász et al., the large majority of the 44 included cohorts reported that 70–80% of episodes were classified as mild, regardless of the severity scale used [9].

Strikingly, across the entire seven-year Canadian series discussed above, just over one in ten episodes (10.6%) met criteria for moderately severe disease, and not a single case in that cohort was classified as severe [13]. This shows a markedly more favorable severity profile than the 8% severe-disease proportion observed in the AP group of the present cohort, despite both studies applying broadly NASPGHAN-based severity criteria. This discrepancy may reflect genuine variation in cases between the two tertiary referral populations, differences in regional referral patterns for complicated pancreatitis, or simply the small absolute number of severe episodes in both studies (which limits the precision of any percentage-based comparison). It underscores the value of future multicenter collaboration to obtain more stable, generalizable estimates of severe-AP prevalence in children.

A recently published systematic review and meta-analysis of pediatric AP-to-ARP-to-CP progression provides further relevant context. Pooling data across multiple cohorts, Gagyi et al. found that the risk of recurrence was significantly influenced by both the etiology and the severity of the initial AP episode, while structural and genetic factors were specifically associated with later progression from ARP to CP [5]. This adds an important nuance to the present findings: although ARP and first-episode AP did not differ significantly in severity distribution in this cohort, the pooled evidence suggests that initial-episode severity may still carry prognostic weight for the subsequent risk of recurrence.

The significantly lower serum albumin observed in moderate/severe disease in the present cohort reproduces one of the most consistent findings in the pediatric severity-prediction literature. In the meta-analysis by Juhász et al., admission albumin showed a non-significant trend toward lower values in moderate/severe compared with mild disease (pooled mean difference −3.34 g/L; 95% CI: −8.20 to +1.52 g/L) [9]. Separately, a limited number of studies within the same review reported that albumin below 34 g/L measured at 48 h demonstrated promising discriminative ability for predicting a complicated disease course, with a pooled sensitivity of 91.0% and specificity of 75.7% across two cohorts [9]. Similarly, the non-significant trends toward higher amylase and higher 24–48-h CRP in the moderate/severe group of the present study are directionally consistent with the same meta-analysis, which found pooled CRP to be significantly higher in moderate/severe disease [9]. The failure of these markers to reach statistical significance in the present cohort most likely reflects the limited number of severe episodes rather than a true absence of association.

### 4.5. Length of Hospitalization and Its Determinants

The median length of hospitalization in the present cohort did not differ significantly between AP and ARP. When episodes were stratified by severity, the median length of hospitalization differed significantly between mild and moderate/severe cases. These median-based figures are directly comparable with the largest available pediatric datasets, most of which likewise report length of stay (LOS) as a median. In a US multicenter cohort of 7693 discharges from the Pediatric Health Information System, median LOS was 4 days (IQR 3–7) [19], closely matching both the present AP-group median and the present mild-disease median. A smaller two-center US cohort reported a shorter median LOS of 3 days, in a population in which idiopathic disease predominated [20]. By contrast, a Bahraini single-center cohort reported a mean LOS of 7.68 days [18]. This length, while not directly comparable in distributional terms, sits numerically close to the present moderate/severe-disease median.

A separate analysis restricted to the single-episode AP subgroup demonstrated that etiological complexity—rather than recurrence status—was a significant determinant of hospitalization length, with patients whose pancreatitis arose from complex, multifactorial, or systemic causes experiencing substantially longer hospital stays than those with idiopathic or single-factor disease, while the latter two groups did not differ meaningfully from each other. This finding is conceptually aligned with the NASPGHAN classification, which explicitly links greater disease severity to prolonged hospitalization as part of its clinical definition [7], and with multicenter US data demonstrating that etiological complexity independently predicts longer stays in children with AP [19]. Taken together, these results support etiological complexity at first presentation—rather than recurrence status alone—as a clinically meaningful early predictor of resource utilization in pediatric AP.

The most relevant findings in the articles discussed above compared with this study’s findings are summarized in Table 4.

### 4.6. Study Limitations

This study is subject to several limitations inherent to its retrospective, single-center design. Data were extracted from existing medical records rather than collected prospectively under a standardized protocol, which carries an inherent risk of incomplete or inconsistently documented variables, particularly for symptoms or laboratory parameters not routinely recorded at every encounter. As a tertiary referral center, our institution likely receives cases of more complex or diagnostically uncertain presentations, which may have inflated the apparent burden of multifactorial and genetic etiologies relative to what would be observed in a general pediatric population. Conversely, genetic testing was not performed uniformly across all patients but was more frequently pursued after a recurrent episode, introducing a potential ascertainment bias that may partly explain, rather than purely reflect, the higher genetic-etiology proportion observed in the ARP group.

From an analytical standpoint, the small number of patients within certain subgroups, most notably the severe-disease category, limited the statistical power available to detect associations involving severity, and the resulting wide confidence intervals should temper any causal interpretation of the non-significant trends reported for amylase and CRP. The absence of a multivariable model adjusting simultaneously for age, sex, etiology, and severity means that the individual associations identified here, including etiological complexity as a determinant of length of stay, cannot be assumed to be independent of one another, and the lack of longitudinal follow-up precluded any assessment of outcomes beyond the observation window.

### 4.7. Study Strengths

The main strength of this study lies in the distinction between what it contributes as novel and what it corroborates. Its genuinely original contribution is the introduction of an etiological-complexity framework—stratifying causes into idiopathic, single-factor, and complex categories—which, to our knowledge, has not previously been applied to pediatric AP cohorts, and which identified etiological complexity at first presentation as a significant predictor of prolonged hospitalization. Several other findings reproduce patterns already established in large international and meta-analytic datasets—the predominance of genetic etiologies in ARP, the association between admission hypoalbuminemia and moderate-to-severe disease, and the predominance of idiopathic disease in first-episode AP; rather than presenting these as new discoveries, we regard them as independent external corroboration of established patterns in a population that has been substantially underrepresented in the literature. This regional dimension is itself a meaningful contribution: the study is, to our knowledge, one of the few systematic characterizations of AP and ARP originating from Central and Eastern Europe, a region whose evidence base has until now been shaped almost entirely by North American, Western European, and East Asian cohorts.

## 5. Conclusions

In this Romanian pediatric cohort, first-episode AP was dominated by idiopathic and infectious causes, whereas genetic etiologies accounted for more than one-third of ARP cases, supporting a stepwise diagnostic approach in which comprehensive etiological work-up, including genetic testing, is reserved for children with recurrent disease. This higher genetic-etiology proportion in recurrent disease should, however, be interpreted in light of the selective, predominantly post-recurrence application of genetic testing, which may have contributed to the observed difference. Two clinically actionable predictors emerged: etiological complexity at first presentation, which identifies children likely to require prolonged hospitalization, and admission hypoalbuminemia, which flags moderate-to-severe disease using a simple, widely accessible laboratory test.

## Figures and Tables

**Table 1:** Baseline demographic and biological markers of children with acute pancreatitis and acute recurrent pancreatitis.

Variable	AP (Median, IQR)	ARP (Median, IQR)	Mann–Whitney (U, *p*)
Age (years)	10.79 (6.9)	11.67 (7.5)	U = 663.0, *p* = 0.902
Hospitalization length (days)	6.00 (3)	4.00 (4)	U = 778.0, *p* = 0.271
Leukocytes (×10^3^/μL)	8.89 (8.81)	7.95 (8.08)	U = 592.5, *p* = 0.936
Lymphocytes (×10^3^/μL)	1.75 (1.44)	1.85 (1.08)	U = 552.0, *p* = 0.583
AST (U/L)	32.0 (20.75)	25.5 (11)	U = 738.0, *p* = 0.112
ALT (U/L)	24.0 (57.25)	15.0 (19.75)	U = 764.5, *p* = 0.058
GGT (U/L)	20.3 (80)	15.5 (11.75)	U = 662.5, *p* = 0.061
Total bilirubin (mg/dL)	0.80 (0.97)	0.79 (1.14)	U = 468.0, *p* = 0.804
Albumin (g/dL)	4.1 (0.83)	4.23 (0.8)	U = 394.0, *p* = 0.981
Triglycerides (mg/dL)	87 (67)	75 (59.5)	U = 498.5, *p* = 0.657
Amylase (U/L)	353 (1084)	447 (311)	U = 656.0, *p* = 0.843
Lipase (U/L)	733 (1286)	755 (941)	U = 537.0, *p* = 0.882
CRP at admission (mg/dL)	0.69 (2.46)	0.83 (3.04)	U = 484.5, *p* = 0.427
CRP 24–48 h (mg/dL)	1.26 (5.27)	2.3 (11.30)	U = 288.0, *p* = 0.443
Procalcitonin (ng/mL)	0.22 (0.75)	0.17 (0.24)	U = 165.5, *p* = 0.595

Abbreviations: AP, acute pancreatitis; ARP, acute recurrent pancreatitis; AST, aspartate aminotransferase; ALT, alanine aminotransferase; GGT, gamma-glutamyl transpeptidase; CRP, C-reactive protein.

**Table 2:** Distribution of the etiologies of acute pancreatitis and acute recurrent pancreatitis in the study population.

Group	Biliary *n* (%)	Genetic *n* (%)	Idiopathic *n* (%)	Infection *n* (%)	Multifactorial *n* (%)	Other *n* (%)	Toxic *n* (%)	Total
AP	8 (16)	1 (2)	12 (24)	7 (14)	7 (14)	9 (18)	6 (12)	50
ARP	4 (15)	10 (37)	1 (4)	0 (0)	2 (7)	8 (30)	2 (7)	27
Total	12 (16)	11 (14)	13 (17)	7 (9)	9 (12)	17 (22)	8 (10)	77

Abbreviations: AP, acute pancreatitis; ARP, acute recurrent pancreatitis.

**Table 3:** Comparison of clinical and biological markers between episodes of different severity.

Parameter	Mild (*n* = 52)	Moderate/Severe (*n* = 25)	Mann–Whitney (U, *p*)
Age (years)	11.29 (7.32)	11.50 (6.50)	U = 657.5, *p* = 0.939
**Hospitalization length (days)**	**5.0 (4.25)**	**7.0 (8.0)**	**U = 396.0, *p* = 0.005**
Leukocytes (×10^3^/μL)	8.24 (7.17)	10.43 (8.60)	U = 587.0, *p* = 0.775
Lymphocytes (×10^3^/μL)	1.89 (1.08)	1.40 (1.73)	U = 721.5, *p* = 0.215
AST (U/L)	32.0 (53.0)	28.0 (10.0)	U = 711.5, *p* = 0.260
ALT (U/L)	25.0 (64.0)	18.0 (17.0)	U = 687.0, *p* = 0.398
GGT (U/L)	19.0 (86.0)	17.5 (12.75)	U = 587.0, *p* = 0.557
**Albumin (g/dL)**	**4.10 (0.83)**	**3.75 (1.13)**	**U = 588.0, *p* = 0.030**
Total bilirubin (mg/dL)	0.65 (1.12)	0.90 (0.73)	U = 425.5, *p* = 0.434
Triglycerides (mg/dL)	82.0 (53.0)	102.0 (122.0)	U = 427.0, *p* = 0.243
Amylase (U/L)	348.0 (479.0)	482.0 (969.0)	U = 486.5, *p* = 0.076
Lipase (U/L)	725.0 (1479.0)	768.5 (981.0)	U = 439.0, *p* = 0.462
CRP at admission (mg/dL)	0.69 (2.57)	1.16 (2.91)	U = 489.5, *p* = 0.304
CRP at 24–48 h (mg/dL)	1.05 (5.27)	2.68 (11.30)	U = 261.0, *p* = 0.092
Procalcitonin (ng/mL)	0.16 (0.75)	0.22 (0.24)	U = 135.0, *p* = 0.206

Abbreviations: AST, aspartate aminotransferase; ALT, alanine aminotransferase; GGT, gamma-glutamyl transpeptidase; CRP, C-reactive protein.

**Table 4:** Comparison of the present cohort with comparable pediatric acute pancreatitis (AP) studies.

Study	Country/Region	Number of Patients	Mild Disease (%)	Leading Etiology	Hospitalization (Days)
Present study—AP vs. ARP	Romania	63 (77 episodes)	72.0 (AP)/59.3 (ARP)	Idiopathic 24.0% (AP)/Genetic 37.0% (ARP)	Median: 6 (AP)/4 (ARP)
Present study—etiological complexity a	Romania	50 (single-episode subgroup)	—	Idiopathic/Single-factor/Complex (compared)	Mean: 5.42/5.96/9.93
Juhász et al. [9]	Multinational (44 cohorts)	Pooled (44 studies)	70–80 (typical)	Variable by cohort/region	NR
Tian et al. [4]	Multinational (48 studies)	Pooled (48 studies)	NR	Gallstones (Asia); Idiopathic (Europe/NA/SA); Trauma (Oceania)	NR
Gagyi et al. [5]	Multinational (meta-analysis)	Pooled	NR	Recurrence/progression risk by etiology & severity	NR
Wang et al. [16]	China (Yunnan–Guizhou)	275 (330 episodes)	≈90 (SAP 10.2%)	Biliary tract disease + viral infection	NR
Zhong et al. [12]	Western China	130	NR	Biliary 31.5%/Idiopathic 28.5%	NR
Korman et al. [13]	Canada (Montréal)	208	89.4 (mod-sev 10.6, severe 0)	Medication 20.2%/Biliary 16.4%/Infection 15.4%	NR
Mroskowiak et al. [14]	Poland	57	NR	Idiopathic 35.1%/Biliary 24.6%/Genetic–anatomical 24.6%	NR
Hou et al. [15]	China	102	NR	Idiopathic 28.4%/Traumatic 25.5%/Biliary 23.5%	NR
Rkain et al. [17]	Morocco	15	NR	Idiopathic 26.6%/Traumatic 20.0%/Biliary 20.0%	NR
Gay et al. [19]	USA (multicenter)	7693 discharges	NR	Not etiology-focused (LOS predictors)	Median: 4 (IQR 3–7)
Shahein et al. [20]	USA (2 centers)	92	≈74 (68/92)	Idiopathic (78%)	Median: 3
Al Hindi et al. [18]	Bahrain	56	NR	Mixed (single-center series)	Mean: 7.68

a Restricted to the single-episode (first AP) subgroup (*n* = 50); patients with acute recurrent pancreatitis (ARP) were analyzed separately (row above). NR = not reported in the original publication. SAP = severe acute pancreatitis; mod-sev = moderately severe acute pancreatitis. For Korman et al. [13], the mild-disease percentage (89.4%) was derived as 100% minus the moderately severe (10.6%) and severe (0%) rates reported in the original publication. IQR = interquartile range; SD = standard deviation. Hospitalization length is reported using the statistic given in the original publication (median with interquartile range [IQR], or mean ± standard deviation [SD]), as indicated in each cell; values were not converted between statistics.

## Data Availability

The original contributions presented in this study are included in the article. Further inquiries can be directed to the corresponding author.

## Abbreviations

The following abbreviations are used in this manuscript:

ALT	Alanine Aminotransferase
ANOVA	Analysis of Variance
AP	Acute Pancreatitis
ARP	Acute Recurrent Pancreatitis
AST	Aspartate Aminotransferase
CI	Confidence Interval
CP	Chronic Pancreatitis
CRP	C-Reactive Protein
CT	Computer Tomography
GGT	Gamma-Glutamyl Transpeptidase
ICU	Intensive Care Unit
INSPPIRE	International Study Group of Pediatric Pancreatitis: In Search for a CuRE
IQR	Interquartile Range
LOS	Length of Stay
MRCP	Magnetic Resonance Cholangiopancreatography
NASPGHAN	North American Society for Pediatric Gastroenterology, Hepatology and Nutrition
SAP	Severe Acute Pancreatitis
SD	Standard Deviation
US	Ultrasound
